# Artemin/GFRA3 axis and TRP channels: molecular insights from a feline model of osteoarthritis

**DOI:** 10.3389/fpain.2026.1716651

**Published:** 2026-03-17

**Authors:** Joshua J. Wheeler, Chie Tamamoto-Mochizuki, Margaret E. Gruen, B. Duncan X. Lascelles, Santosh K. Mishra

**Affiliations:** 1 Department of Molecular Biomedical Sciences, College of Veterinary Medicine, North Carolina State University, Raleigh, NC, United States; 2 Comparative Medicine Institute, North Carolina State University, Raleigh, NC, United States; 3 Department of Small Animal Clinical Sciences, College of Veterinary Medicine, University of Tennessee, Knoxville, TN, United States; 4 Department of Clinical Sciences, College of Veterinary Medicine, North Carolina State University, Raleigh, NC, United States; 5 Translational Research in Pain, College of Veterinary Medicine, North Carolina State University, Raleigh, NC, United States; 6 Comparative Pain Research and Education Centre, Department of Clinical Sciences, College of Veterinary Medicine, North Carolina State University, Raleigh, NC, United States; 7 Center for Translational Pain Research, Department of Anesthesiology, Duke University, Durham, NC, United States; 8 Thurston Arthritis Center, UNC, Chapel Hill, NC, United States

**Keywords:** artemin, osteoarthritis, pain, sensory neurons, TRP channels

## Abstract

**Introduction:**

Degenerative Joint Disease (DJD) is a form of highly prevalent osteoarthritis in humans and animals, including cats, which causes significant pain and hypersensitivity. Despite its prevalence, the mechanisms underlying the DJD-associated pain in cats are poorly understood. While transient receptor potential (TRP) ion channels are expressed in the dorsal root ganglia (DRG) and are implicated in osteoarthritis pain (e.g., through Artemin/GFRA3-mediated changes to TRPV1 and TRPA1 electrical properties), there is currently only indirect evidence of TRP ion channel expression in the feline DRG. This study aims to address this knowledge gap.

**Methods:**

Fura-2 based *in vitro* calcium imaging was used to confirm the functional expression of TRPV1, TRPV2, TRPA1, and TRPM8 in healthy cat DRG neurons. A quantitative reverse transcription-polymerase chain reaction (qRT-PCR) with SYBR green was used to confirm and compare mRNA expression of pain sensors including TRPV1, TRPV2, TRPV4, TRPA1, TRPM3, TRPM8, MRGPRD, TAC1, and GFRA3 in the DRG neurons of healthy cats and DJD group. Finally, serum artemin concentrations were quantified using enzyme linked-immunosorbent assay (ELISA) and compared between healthy and DJD cats.

**Results:**

Functional responses of TRPV1, TRPV2, TRPA1 and TRPM8 were determined via calcium imaging in DRG neurons obtained from healthy cats. Gene expression is further extended into healthy vs. DJD cats. While TRPV1, TRPV2, and TRPM8 showed a >1.5-fold increase in cats with DJD compared to healthy controls, MRGPRD mRNA expression showed a corresponding ∼1.5-fold decrease. However, these increases or decreases in fold-change did not reach statistical significance. GFRA3, a receptor for artemin, was found to be expressed in the cat DRG, though its levels remained unchanged in DJD-affected cats. Lastly, a significant association was found between serum artemin concentrations and radiographic DJD scores but not with veterinarian pain scores.

**Discussion:**

Our findings characterize functional expression of several pain and hypersensitivity-associated TRP ion channels in cat DRG neurons and identify the Artemin/GFRA3/TRP axis as a potential driver of chronic pain. The expression of channels, including TRPV1, TRPA1, and TRPM8 modulated by Artemin/GFRA3 pathway was confirmed. Bridging these cellular findings to DJD state, the observed correlation between serum artemin concentrations and radiographic DJD scores further implicates this pathway in disease severity. These results provide potential early evidence that the Artemin/GFRA3/TRP axis drives pain in feline DJD. This conservation is consistent with findings in other species, such as rodents and canines, suggesting translational relevance for therapeutic targeting.

## Introduction

Degenerative joint disease (DJD), also referred to as osteoarthritis, occurs in upwards of >90% of cats (1, 2). DJD in cats is the leading cause of decreased mobility, decreased activity, changes in mood and behavior, changes in sociability, and pain in cats (1, 3, 4). Further, roughly 45% of cats with DJD present with clinically detectable DJD-associated pain (5). Despite the high prevalence of DJD in cats, there are no effective treatments for DJD-associated pain (6, 7). Furthermore, DJD etiology closely mirrors DJD in humans (8); therefore, understanding how DJD develops in cats is likely to assist with identifying novel therapeutics for targeting pain and inflammation associated with DJD in humans, along with helping to understand the natural progression of healthy joints into degenerative joints. Other fields of neuroscience have begun to use naturally occurring neurological diseases in cats as pre-clinical models of naturally occurring human diseases as well (9, 10).

Nociception in mammals, including cats, relies heavily on transient receptor potential vanilloid (TRPV), transient receptor potential ankyrin (TRPA), and transient receptor potential melastatin (TRPM) channels. In particular TRPV1, TRPV2, and TRPM3 are responsible for detecting noxious heat (11–14); TRPM8 and TRPA1 are responsible for detecting noxious cold (15–17); and TRPV4 detects osmotic and pressure changes (18–20). TRPV4 has a demonstrated role in osteoarthritis pain (21). TRPA1 also mediates noxious chemical pain and possibly mediates mechanical pain (22–24). Noxious mechanical pain is mediated by Mas-related G protein receptor D (MRGPRD) (25). Further, Substance P (Tac1) is released from nociceptive neurons to transmit pain sensations from DRG neurons to spinal cord interneurons (26, 27), including in cats (27). Single cell RNA-Sequencing performed in mice indicates that all of these genes are likely to be co-expressed with GDNF (Glial cell line-Derived Neurotrophic Factor) Family Receptor alpha-3 (GFRA3) (28, 29).

Previous work has demonstrated that GFRA3 and its ligand artemin might play a role in osteoarthritis pain (30) in dogs (31), another companion animal. Other researchers have found that artemin can sensitize neurons innervating the bone, including bones found in the joints, to cause pain (32–34). GFRA3 is expressed in roughly 20% of mouse dorsal root ganglion neurons (35, 36) and co-localizes with TRPA1 (37), TRPV1 (37–40), and TRPM8 (41). GFRA3 has been shown to mediate cold allodynia in mice (41, 42) and recruit heat sensitive neurons post-injury (43). GFRA3 is activated after its ligand artemin binds (44, 45) leading to sensory neuron sensitization. Taken together, this research indicates that the Artemin/GFRA3 axis might play a role in sensitization and/or pain in cats with DJD, likely through TRP ion channels as has been demonstrated in mice (37, 42).

Despite their established roles in other species, direct evidence linking the role of these Artemin/GFRA3/TRP channels in cats with and without DJD is currently lacking. While these pathways have been explored in mice and dogs, the functional expression of pain-associated TRP channels in healthy cats remain unknown. Initially, functional expressions of TRPV1, TRPV2, TRPA1, and TRPM8 were demonstrated in cultured DRG sensory neurons from naïve cats using calcium imaging in response to specific TRP channel ligands. Next, to address this knowledge gap, banked tissue samples from clinically characterized healthy cats and cats with DJD were utilized. Serum artemin concentrations were quantified and compared between healthy cats and DJD group, alongside the confirmation of mRNA expression of several pain associated genes in the dorsal root ganglia (DRG) including TRPV1, TRPV2, TRPV4, TRPA1, TRPM3, TRPM8, MRGPRD, Tac1 (Substance P), and GFRA3. These results demonstrate mechanistic foundation for targeting this Artemin/GFRA3/TRP axis in feline chronic pain.

## Materials and methods

### Sample collection and evaluation of Cat DJD scores and pain scores

This study used samples and data collected previously by researchers in the Translational Research in Pain (TRiP) Program at North Carolina State University. All data were collected as approved by North Carolina State University's College of Veterinary Medicine Institutional Care and Use Committee under IACUC protocols 06-056, 08-124, 08-125, 11-102, 14-009, 14-043. Procedures were carried out under informed, written owner consent. The work in the original studies involved the use of non-experimental client-owned animals only and followed established internationally recognized high standards of individual veterinary clinical patient care.

Samples (separate to those required for the individual studies) were collected during previous studies using client owned cats (46–48). Pain scores were determined by clinicians using the Feline Musculoskeletal Pain Index (FMPI) by qualified veterinarians at the North Carolina State University College of Veterinary Medicine (46, 49). Pain was determined using the following scoring system: 0 = no resentment to manipulation; 1 = mild withdrawal, mild resistance to manipulation; 2 = moderate withdrawal, body tenses, may orient to site being examined, may vocalize or increase vocalization; 3 = orients to site, forcible withdrawal from manipulation, may vocalize, hiss, or bite; and 4 = tries to escape or prevent manipulation, bites or hisses, marked guarding of site. Total pain scores were determined by summation of the pain scores of the individual joints on a given cat. Total DJD Scores were determined solely by co-author (BDXL) using radiographs as described previously (2). Following these examinations, cats were determined to either be described as ‘Healthy Cats’ or ‘Cats with DJD’. Healthy Cats had total pain scores ≤4 and total DJD scores ≤12, meanwhile, Cats with DJD had pain scores ≥5 and total DJD scores ≥13 (48). All cats from which samples were collected were evaluated for the presence of other systemic diseases.

To achieve a reasonable power for this study, we had to include samples from cats that had chronic kidney disease (Table 1). Every effort was made to age and sex matched cats that samples were pulled from; however, as DJD is a highly prevalent chronic disease in cats, the ages of the cats were significantly different (Table 2).

**Table 1:** Demographics of the cats used in this study.

Parameter	Number
Breed	
Domestic short hair	54
Domestic medium hair	3
Domestic long hair	5
Maine coon	3
Siamese	2
Rex	1
Himalayan	1
Persian	1
Manx	1
Grey tabby	1
DJD present	
Yes	50
No	25
Pain present	
Yes	50
No	22
Chronic kidney disease diagnosis^a^	
Yes	20
No	31
Sex	
Male	7
Male—neutered	35
Female	4
Female—neutered	26

^a^ Only known for cats used in serum artemin concentration experiment.

**Table 2:** Comparison of demographic parameters between healthy cats and cats with DJD.

Parameter	Healthy cat mean ± SD	DJD cat mean ± SD	Significance^d^
Age	6.23 ± 4.00	12.41 ± 4.17	<0.0001
Weight^a^	4.33 ± 1.58	5.18 ± 1.66	ns
BCS^b^	5.04 ± 1.80	5.30 ± 1.90	ns
DJD score	1.08 ± 6.96	26.95 ± 15.74	<0.0001
Pain score^c^	1.71 ± 2.02	14.68 ± 7.64	<0.0001

^a^ In kg.

^b^ Body condition score.

^c^ Only known for cats used in serum artemin concentration experiment.

^d^ determined using a 2-tailed Welch's *t*-test.

### Serum collection and artemin quantification

Whole blood was collected into a sterile 3 mL anti-coagulant free plastic tube (red top Vacutainer®) and allowed to clot at room temperature for 30–60 min. Clotted samples were centrifuged at 1163 x *g* for 10 min, and serum was removed, aliquoted, and stored in cryovials at −80 °C until use. Cats were not anesthetized during blood collection. A total of 54 serum samples were run: n = 13 healthy (no DJD), *n* = 8 low DJD, *n* = 5 moderate DJD, and *n* = 28 high DJD. Further, *n* = 9 were healthy (no pain), *n* = 10 had low pain scores, *n* = 5 had moderate pain scores, and *n* = 30 had high pain scores. Serum artemin was quantified using a human artemin ELISA kit (Abcam, ab215416), because there are no cat-specific ELISA kits designed to detect cat Artemin. This kit was opted based on high homology between the two proteins (87.2% homology between cat artemin [XP_023114384.1] and human artemin [NP_001129687.1) (Table 3). We tested this validity of a human Artemin by pre-treating feline serum samples with anti-artemin antibody (Thermofisher, PA5-47350; epitope homology is 98% identical between recognized immunogen and cat artemin sequence [XP_023114384.1) or compare with goat-IgG isotype control (CalBioChem, N102-100ug).

**Table 3:** Homology between cat ARTN and common species found in research.

Species	NCBI accession number	Homology (%)	Commercially available kit?
Cat	XP_023114384.1	100	No
Human	NP_001129687.1	87.2	Yes
Rhesus macaque	XP_021796316.1	86.2	Yes
Dog	XP_038414275.1	79.6	Yes
Pig	XP_020952497.1	95.9	No
Rat	NP_445849.1	74.2	Yes
Mouse	NP_001271120.1	74.2	Yes
Zebrafish	XP_005170818.3	48.6	Yes

### DRG isolation and qRT-PCR

DRG isolation and preparations for tissue banking for qRT-PCR samples are described in a previous study (50). Banked L5 DRG from the left side of healthy and severe DJD (x-ray scores ≥11) cats stored in TRIzol were isolated by combining first adding 500 μL chloroform to each sample. Then each sample was vortexed for 30 s and then homogenized for 45 s. Samples were then allowed to sit at room temperature for 5 min. The aqueous layer, containing the mRNA, was then moved to a separate 1.5 mL Eppendorf tube. RNA was isolated from the aqueous layer using QIAgen's RNeasy Fibrous Tissue Mini kit without a Proteinase K digestion. For total RNA isolation and cDNA synthesis, previously published protocol utilized for mouse (51) and dog (31, 52) DRGs. In total there were 18 L5 DRG from 9 healthy and 9 DJD cats. The demographics of these cats are in Tables 1, 2.

SYBR Green primers for cat *TRPV1, TRPV2, TRPV4, TRPA1, TRPM3, TRPM8, MRGPRD, TAC1,* and *GFRA3* were designed using NCBI's Primer3 and BLAST (53). The sequences used for each primer were found in Table 4. Each primer set was aligned to genes from the domestic house cat (*Felis catus*, organism ID: 9685). To determine changes in expression of these genes between the healthy and DJD cats, the Pfaffl method (54) was used. To ensure accurate quantifications of mRNA transcripts, efficiency was determined using primer sets to generate a standard curve from a pool of cDNA (2 DJD and 2 healthy cats). The stock concentration was measured via spectrophotometry (NanoDrop) and adjusted to 500 ng/µL. A series of 1:1 serial dilution series was then created to produce eleven additional concentrations: 250, 125, 72.5, 36.25, 18.125, 9.0625, 4.5, 2.27, 1.13, 0.57, and 0.28 ng/µL. Since 1:1 dilutions were used, Efficiency (E) was calculated using Equation 1 and the fit data is shown in (Supplementary Figure S1).

(1) $$E=2^{-1/Slope}$$

**Table 4:** Information on the probes developed and used for the SYBR green experiments in this work.

Gene	NCBI accession ID	Forward primer (5’ → 3’)	Reverse primer (5’ → 3’)	T_m_ (°C)	Primer efficiency (E)
*TRPV1*	XM_023244720.1	GCAGTGACACCCCCATTCAA	CCACGTACTCCTTGTGGTGG	81.83	1.581
*TRPV2*	XM_0198117212.2	TGGAAGAACAGAGACCTCCAGA	GCACCTTTCGGAAGCCAGAG	81.23	1.747
*TRPV4*	XM_023241517.1	CACCCGTGAGAACACCAAGT	GAAAGACCCCAATCTTGCCG	81.09	6.645
*TRPA1*	XM_011291355.3	ACACATCCCCTTTGCACCAT	GAGGTTTGGGTTGGCTCCTT	80.03	1.522
*TRPM3*	XM_019815753.1	AAACACAAGATGCCTGCCGT	CATCGCAGACAACAACTGGC	82.85	2.273
*TRPM8*	XM_006935704.4	TGTCATTTCCGTGACCGGAG	TCTCTCACCACTTCCCCGAT	83.47	1.641
*MRGPRD*	XM_023240174.1	ATCTGGTACAAGCGTCACCG	GGAGTCCGTCACGAAACACT	82.52	2.724
*GFRA3*	XM_003980803.5	CTCTGGTAGCTGGACTTCCC	GTCAGTTTCAACGATGGCCC	82.72	2.226
*TAC1*	XM_003982791.6	GGAATCCTTCGGGCCTGC	AAGATGCTCAAAGGGCTCGG	80.95	1.617
*GAPDH*	NM_001009307.1	GAAGGTCGGTGTGAACGGAT	TTCCCGTTCTCCAGCCTTGAC	82.42	1.660

Efficiencies were calculated in Equation 1 in Equation 2 to evaluate the relative expression of each gene for each individual sample [see Equation 2 in (54)]. The efficiencies were also used to calculate the changes in gene expression between DJD cats and Healthy cats [R_aiff_, see Equation 1 in (54)].

All samples were run on a Step OnePlus real-time PCR system and the data were analyzed using the Applied Biosystems StepOnePlus software version 2.3. Each sample was run in triplicate (3× technical replicates). Each technical replicate contained the following: 10 μL *Power*SYBR® Green PCR Master Mix (Applied Biosystems, 4367695), 1 μL cDNA, 1 μL Primer set (Forward and Reverse primers were set to a concentration of 10 μM), and 8 μL ddH_2_O. Non-transcript controls contained no cDNA, 9 μL ddH_2_O, and 10 μL *Power*SYBR® Green PCR Master Mix. Melt curves for all technical replicates and biological replicates were collected. *GAPDH* was used as our housekeeping gene based on our previous work in dogs and mice (31, 51, 52). No correlations were observed between the cycle threshold, C_T_, of *GAPDH* and Age, body condition score (BCS), x-ray scores, body weight, and DJD status (Supplementary Table S1) indicating that GAPDH expression is not affected by these variables in our samples and can be used as a housekeeping gene.

### DRG cell culture and calcium imaging

Feline dorsal root ganglia (DRG) were isolated and dissociated into single-cell suspension following a modified protocol (55). Briefly, DRGs (L3–L5) were harvested from a feline cadaver immediately postmortem and placed in Hank's balanced salt solution (HBSS; without Ca^2+^/Mg^2+^). For enzymatic dissociation, tissue was incubated in dissociation medium containing collagenase type III (10 mg/ml) and dispase (5 U/ml) at 37°C for 60 min, with gentle trituration every 15 min using a Pasteur pipette, followed by additional trituration with a fire-flamed pipette. After centrifugation (1,000 rpm, 5 min), the pellet resuspended in 0.05% trypsin in PBS and incubated at 37°C for 30 min with intermittent trituration. The enzymatic reaction was terminated with complete medium [DMEM supplemented with 10% FBS and 1% penicillin–streptomycin], followed by centrifugation. The supernatant was removed, leaving 500 μl, and the pellet was resuspended. To remove debris, the suspension was carefully layered onto 15% FBS in DMEM and centrifuged at 1,000 rpm for 15 min. The debris-containing layer was discarded, and the final pellet was resuspended in 100 μl of complete medium. Cell viability was assessed by trypan blue exclusion. Approximately 20 μl of cell suspension was seeded onto poly-L-lysine/laminin–coated coverslips in 12-well plates, incubated at 37°C in 5% CO_2_ for 1.5 h to allow attachment, and subsequently overlaid with 1 ml of complete medium. Cultured neurons were maintained overnight at 37°C in 5% CO_2_.

Calcium imaging was performed using the method previously described (51, 52, 56): After the overnight incubation at 37 °C in 5% CO₂, cells were loaded with Fura-2AM (1 mg/mL) and placed back into the cell incubator at 37°C in 5% CO₂ for 30 min. Calcium imaging was performed on a Nikon TE200 microscope. During imaging, the cells were kept alive by perfusing the coverslip with Locke Buffer. Cells were continuously perfused with Locke buffer during the duration of the experiment. A minimum of 90 s was used to ensure that each compound was fully washed out between agonist applications. Cells were sequentially exposed to 100 µL of the following solutions: 100 µM icilin (15), 100 µM allyl isothiocyanate (AITC) (22, 23), 100 µM Probenecid (57), 1 µM capsaicin, and 100 mM KCl. Positive responses were defined as a fluorescence increase of A_340_/A_380_ > 0.1, with analysis limited to neurons confirmed by their responsiveness to terminal application of 100 mM KCl. Cells responding to these agonists and which responded to KCl were counted and normalized to the total number of cells responding to KCl.

### Statistical analysis

**Figure 1:**
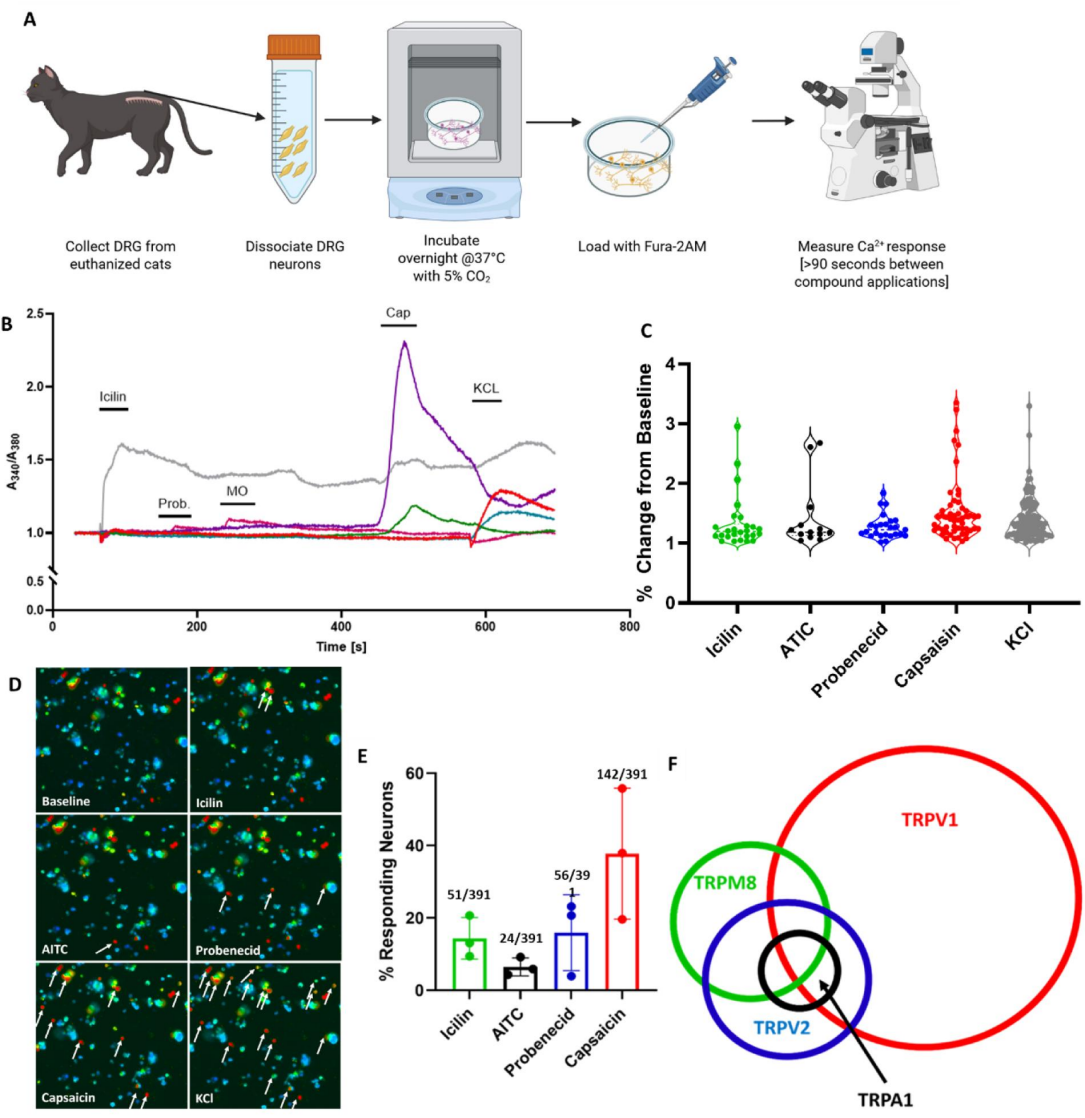
Functional characterization of selected TRP channels on cultured DRG sensory neurons from healthy cats. **(A)** Cartoon schematic of the process of calcium imaging using cat DRG. **(B)** Representative traces of cells responding to Icilin (100 µL of a 100 µM solution), Probenecid (100 µL of a 100 µM solution) [Prob.], AITC (100 µL of a 100 µM solution) [MO], Capsaicin (100 µL of a 1 µM solution), or KCl (100 µL of a 100 mM solution). A minimum of 90 s elapsed between initial application and application of the next compound. **(C)** Violin plot of the responses to each compound. Each dot represents 1 neuron. **(D)** Representative false color images of neurons at baseline and following application of Icilin, AITC, Probenecid, Capsaicin, and KCl. **(E)** Percentage of cat DRG sensory neurons responding to icilin (TRPM8 agonist, 100 µM), ally isothiocyanate (TRPA1 agonist, 100 µM), probencid (TRPV2 agonist, 100 µM), and capsaicin (TRPV1 agonist, 1 µM). Responses were normalized to 100 mM KCL. Values above each bar indicate the ratio of the agonist-induced response to the potassium chloride (KCl) calcium transients (Numerator: Agonist/Denominator: KCl). Each dot represents one biological replicate. **(F)** Venn Diagram showing the overlap of differently responding DRG sensory neuron populations. Data is presented as Mean ± Standard Deviation. No statistical tests were performed for this figure. Created in BioRender. Wheeler, J. (2026) https://BioRender.com/q373vah.

**Figure 2:**
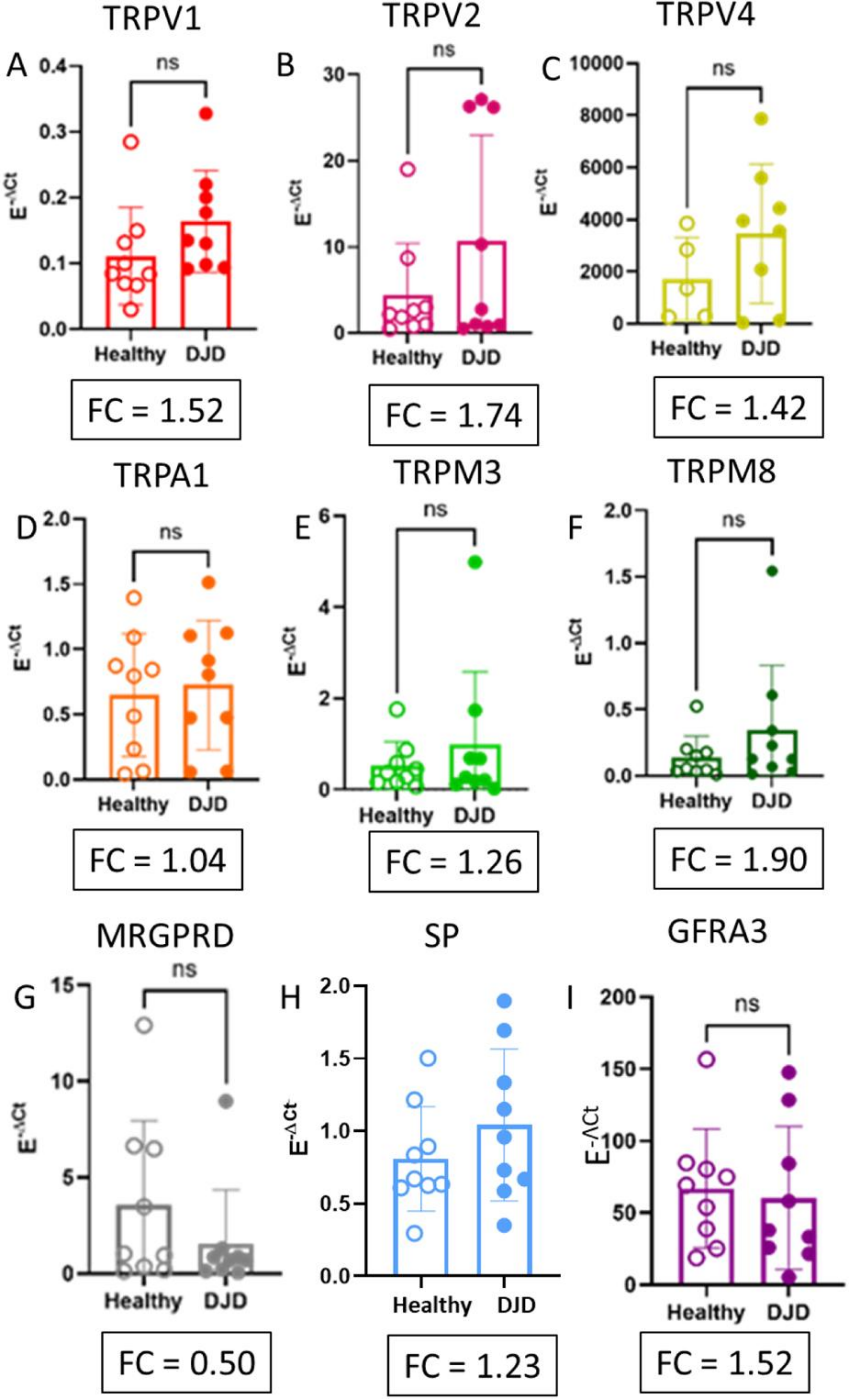
SYBR green qRT-PCR results for TRP channels and substance P expressed in the DRG from healthy and DJD + cats. **(A–H)** Individual results for **(A)** *TRPV1*, **(B)** *TRPV2*, **(C)** *TRPV4*, **(D)** *TRPA1*, **(E)** *TRPM3*, **(F)** *TRPM8*, **(G)** *MRGPRD*, **(H)** TAC1 [Substance P (SP)], and **(I)** *GFRA3*. No significant differences were seen between Healthy and DJD + cats; however, the Fold Changes (FC) calculated with the Pfaffl Method show an FC increase in expression of TRPV1, TRPV2, and TRPM8 mRNA. Data is presented as Mean ± SD, *n* = 9 for both sample groups. Each dot represents one biological replicate. Significance in all panels was determined using a Mann–Whitney *U*-Test, ns, not significant.

Graphpad 8.3 (Prism) was used for statistical analysis. A Mann–Whitney *U* Test was used to test for significance in Figures 1, 2. A Simple linear regression was also used to determine the correlation between serum artemin concentrations and DJD scores or total Pain scores in Figure 1 and for the other correlations (Supplementary Figure S5). Specifics of which test were used are found in the figure legends.

Body condition score (BCS), x-ray scores, Total DJD Scores, and Pain Scores were treated as continuous variables for both the ELSIA and qRT-PCR experiments as these metrics function as a Likert scale with ≥5 possible bins (58–61).

For the qRT-PCR experiments, nine cats per health group were selected based on previous work from our lab in dogs (31) and mice (51, 62) where a minimum of 9 biological replicates were needed to detect significant differences. These were based on using the recommended power (1—*β*) = 0.08, and *α* = 0.05 (63).

## Results

### Functional TRP expression in healthy cats

Functional expressions of TRPV1, TRPV2, TRPA1, and TRPM8 were examined via *in vitro* Fura-2 calcium imaging in cultured neurons from healthy cat DRG (Figure 1). These specific molecules were selected for analysis based on their established roles as peripheral pain sensors. To first confirm that these genes were functionally expressed in DRG sensory neurons from healthy cats, calcium transients were measured in response to specific ligand to these receptors. Around 14.4 ± 5.8% of sensory neurons responded to icilin, a TRPM8 agonist (15); 6.5% ± 2.5% responded to allyl isothiocyanate (AITC), a TRPA1 agonist (22); 15.9% ± 10.5% responded to probenecid, a TRPV2 agonist (57); and that 37.8% ± 18.1% responded to capsaicin, a TRPV1 agonist (11) (Figure 1B). Fura-2 calcium imaging enables sequential testing of multiple agonists that allows to build a population map of these DRG neurons (Figure 1C).

### Differential gene expression between healthy and DJD cats

To confirm the expression of DRG-expressed pain-associated TRP ion channels in cat DRG. GFRA3 has been demonstrated to upregulate expression of several of these TRP ion channels (37). mRNA expression of TRPV1, TRPV2, TRPV4, TRPA1, TRPM3, TRPM8, along with MRGPRD, and Tac1 (Substance P) (Figure 2) was measured using Using SYBR Green qRT-PCR. No significant differences in mRNA expression of these genes were detected between healthy cats and cats with DJD; however, fold change (FC) analysis revealed that TRPV1, TRPV2, and TRPM8 exhibited FC > 1.5, suggesting a trend toward increased expression in the DRG of cats with DJD. Conversely, MRGPRD showed an FC < 0.5, indicating a downward trend in expression. Despite these shifts, neither the increases nor decreases reached statistical significance. Given that artemin binds to GFRA3, receptor for artemin (44, 45), GFRA3 mRNA expression was evaluated in L5 DRG to determine if DJD drove receptor upregulation. Consistent with other targets, no significant changes in GFRA3 mRNA were observed between groups (Figure 2I).

### Circulating artemin concentration correlates with radiographic DJD severity

To investigate artemin as a systemic biomarker in the absence of a feline-specific assay, the validity of a human artemin ELISA was first established for use in feline serum. Specificity of ELISA was validated by neutralization assay—the addition of dose-dependent increase in neutralizing anti-ARTN antibody resulted in a decrease in detectable Artemin (Supplementary Figure S2), indicating successful sequestration of the feline protein. Although the magnitude of sequestration varied between individual feline samples—reaching a maximum reduction of approximately 60% at the highest antibody dose (64 µg/mL), the consistent downward trend across samples confirms ELISA's specificity.

**Figure 3:**
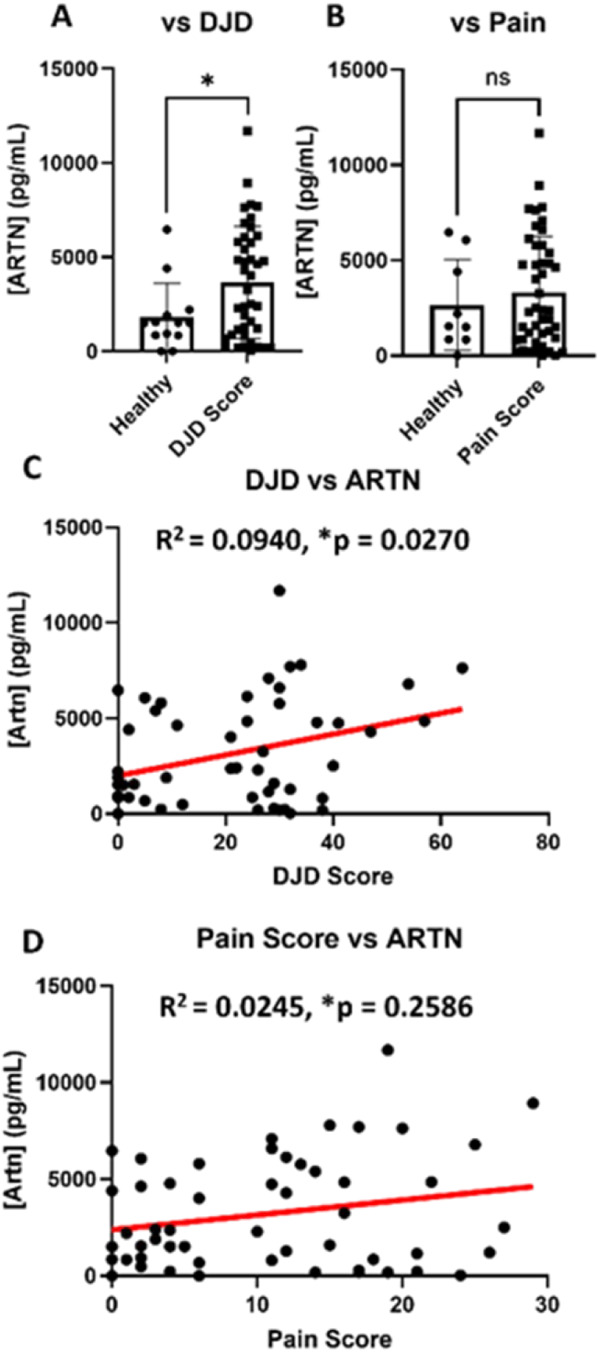
Serum artemin is significantly increased in patients with quantifiable DJD scores. **(A)** Serum concentrations of artemin are significantly increased (*p = 0.0109) in cats with DJD. **(B)** Serum concentrations of artemin are not an indicator of pain in cats with DJD. **(C)** Serum artemin concentration significantly correlates with DJD scores. **(D)** Serum artemin concentration does not correlate with pain scores in cats with DJD. Data is presented as Mean ± SD, Significance was determined using Welch's *t*-test for **(A,B)**, ns, not significant. Correlations in **(C,D)** were determined using a simple linear regression. Each data point represents one biological replicate.

Given that Artemin concentrations are positively correlated with negative outcomes in dogs (31), this study first sought to determine if serum artemin concentrations were increased and/or correlated with radiographic DJD scores. A significant increase in serum artemin concentrations was found in cats based on their respective radiographic DJD scores (Figure 3A). To our surprise, artemin levels remain insignificant compared to subjective pain scores (Figure 3B). This pattern was reflected in correlation analysis: there was a weak, but significant, correlation between serum artemin concentration and radiographic DJD score (Figure 3C), but no significant correlation between serum artemin concentration and pain scores (Figure 3D). To confirm whether these findings were specific to joint pathology or not, potential systemic confounds were analyzed. As chronic kidney disease (CKD) is a common comorbidity in aging cats, serum artemin was compared in healthy cats vs. those with CKD; no significant difference was observed (Supplementary Figure S3). Further multivariate correlation confirmed that while CKD had a minor effect on Artemin levels, it did not negate the primary relationship with DJD (Supplementary Figure S4). Lastly, to determine whether systemic artemin levels were influenced by general physical characteristics, a linear regression analysis was performed against age, body weight, and body condition score (BCS). Serum artemin concentrations were not significantly correlated with any of these parameters (Supplementary Figure S5), suggesting that the observed elevations are independent of a cat's age or physical size.

## Discussion

Expression and functional activity of TRPV1, TRPA1, TRPV2, and TRPM8 in the feline DRG—alongside the novel identification of TRPM3 mRNA was confirmed—establishes the cellular machinery necessary for Artemin-mediated hypersensitivity in cats (Figures 1, 2). Furthermore, expression of genes previously associated with the Artemin/TRP axis in healthy and feline osteoarthritis were established, identifying artemin as a potential systemic biomarker for radiographic DJD severity (Figure 3). These results provide a mechanistic foundation for targeting the GFRA3/TRP pathway to manage chronic pain.

### Artemin as a putative biomarker for disease severity

Prior research in dogs has found a correlation between increased serum artemin concentrations and decreased limb use in dogs (31), but not between serum artemin and hypersensitivity in dogs (64). Increased serum artemin concentrations were found in human patients with osteoarthritis; however, this increase was not statistically significant (31). Our results demonstrate a weak, but significant, correlation between serum artemin concentration and radiographic DJD score of *R*^2^ = 0.0940. Our results suggest artemin as a potential marker for joint degeneration seen in cats with DJD. Although this study represents a foundational step in identifying the role of artemin in joint pathology, and the results should be interpreted as preliminary in nature. Further large-scale, longitudinal studies are warranted to fully validate serum artemin as a clinical/pain biomarker for joint pathology in cats.

### Comparative functional expression/validation of TRP channels in cat DRG

The mRNA expression of TRPV1, TRPV2, TRPV4, TRPA1, TRPM8, and TRPM3—the latter being a novel finding in cat DRG—along with MRGPRD and TAC1 (Substance P) [(27); Figure 2] was confirmed. These findings validate prior indirect evidence based on neuronal responses to various thermal, chemical, and mechanical stimuli in cats (20, 21, 66–69).

TRP mRNA expression levels remained statistically unchanged. Upregulation (FC > 1.5) of TRPV1, TRPV2, and TRPM8 in the DRG of cats with DJD, identifying molecular substrates for altered thermal hypersensitivity: the increased TRPV1 mRNA likely contributes to the reported heat hyperalgesia in DJD cats (70), a conserved symptom across multiple species (37, 71, 72). Upregulation of TRPM8 suggests a predisposition to cold hyperalgesia common DJD feature in humans, dogs, and mice (72, 73). Given TRPM8's role in cold hyperalgesia in mouse models (41, 42), our findings support the presence of a similar cold sensitivity mechanism in feline DJD. Increased in TRPV2 expression is notable, as this channel mediates noxious stretch responses (74) and is upregulated in inflammatory states (75). We hypothesize TRPV2 may encode pain associated with the reduced joint mobility and stretching characteristic of feline DJD (65). Furthermore, the increased TRPM3 mRNA, which responds to noxious heat (14), could be contributing to thermal sensitivity. This upregulation may be a homeostatic response to inflammatory G*_βγ_* subunit activity (76) or reflect an attempt by sensory neurons to modulate the joint environment via TRPM3-derived microRNAs (45, 77). Because these experiments were performed using DRG sensory neurons isolated from patients, further work is needed to confirm our results because they could be spurious due to the limited sample size.

Functional activation of the studied TRP ion channels (TRPV1, TRPA1, TRPM8, and TRPV2) was confirmed by observing robust responses to their respective agonists. Our capsaicin calcium imaging results demonstrate that the percentage of cat DRG neurons expressing functional TRPV1 is similar to that reported in mice (51, 57, 78) and dogs (52).

MRGPRD is not a TRP channel; however, MRGPRD is responsible for mediating noxious mechanical stimuli (25). The decrease in MRGPRD mRNA expression in L5 DRG is an intriguing inverse finding (Figure 2). As MRGPRD neurons innervate the joint (79), this decrease could serve a protective mechanism in severe DJD by limiting certain mechanical hypersensitivity signals. While sample size is an inherent limitation in this clinical research, these findings provide a first map of these transcriptional changes. In the future, longitudinal large-scale studies will be warranted to definitively link these gene expression patterns to modality-specific pain sensitivities.

### Species-specific differences

Despite the significant increase and correlation between serum artemin concentration and total DJD score, GFRA3 mRNA expression remains unchanged in the DJD compared to healthy cats. Previous work from our group did find a significant increase in the mRNA expression of GFRA3 in the ipsilateral DRG of dogs with unilateral osteoarthritis (31), indicating that DRG neuron GFRA3 expression in DJD has some species specific mechanisms.

Our calcium imaging findings indicate species-specific differences for TRPA1, TRPV2, and TRPM8 channels. Fewer cat DRG neurons responded to AITC (a TRPA1 agonist) and probenecid (a TRPV2 agonist) compared to previously published mouse calcium imaging results (51, 57). Our calcium imaging results demonstrated functional TRPM8 expression in approximately 20% of cat DRG neurons. This figure is comparable to immunohistochemistry studies in rats [≈20%; (15) but higher than that reported in mice [≈10%; (16)], further underscoring likely species-specific differences. DRG tissue from cats with confirmed DJD was not available for this analysis; therefore, these findings represent the functional profile of TRPA1, TRPM8, TRPV1, and TRPV2 in naïve feline sensory neurons. Further work is needed to determine if the functional expression is altered in cultured DRG sensory neurons isolated from cats with DJD.

### Study species differences and inverse regulation

Mechanistic differences were evident compared to rodent models. No increase in TRPA1 mRNA was found in the feline DRG [unlike mice, (37)], and calcium imaging showed a lower percentage of TRPA1-responsive neurons compared to mice (51), suggesting a species-specific difference in TRPA1's role in spontaneous feline DJD. Conversely, the decrease in MRGPRD mRNA expression in L5 DRG is an intriguing inverse finding. As MRGPRD neurons may innervate the joint (79), this decrease could serve a protective mechanism in severe DJD by limiting certain mechanical hypersensitivity signals.

### Comparative functional expression/validation of TRP channels

Functional activation of these studied TRP ion channels (TRPV1, TRPA1, TRPM8, and TRPV2) were confirmed by observing robust responses to their respective agonists. Our capsaicin calcium imaging results demonstrate that the percentage of cat DRG neurons expressing functional TRPV1 is similar to that reported in mice [(51, 57); Lai et al., 2021] and dogs (52). In contrast, our findings indicate species-specific differences for other channels. Fewer cat DRG neurons responded to AITC (a TRPA1 agonist) and probenecid (a TRPV2 agonist) compared to previously published mouse calcium imaging results (51, 57). Regarding TRPM8, while the use of icilin as a quantitative assay has not been previously reported [(15)], our calcium imaging results demonstrated functional TRPM8 expression in approximately 20% of cat DRG neurons. This figure is comparable to immunohistochemistry studies in rats [≈20%; (15); Kobayashi et al., 2005] but higher than that reported in mice [≈10%; (16)], further underscoring likely species-specific differences.

### Limitations and potential confounds

While our finding suggests feline Artemin/GFRA3/TRP axis involvement in feline OA, but several limitations were recognized. One of the primary limitations is the methodology, which consists of lack of feline-specific artemin ELISA kit. While the magnitude of sequestration varied between individual feline samples (Supplementary Figure S2)—reaching a maximum reduction of approximately 60% at the highest antibody dose—the consistent downward trend across samples confirms the assay's specificity. Additionally, the acquisition of feline DRG due to the limited availability of cadaveric tissue limits the expression study; therefore, samples were utilized from patient populations for qRT-PCR and serum artemin concentration experiments. Next, samples obtained from patient populations tend to have more than just one chronic disease occurring, which can add unknown clinical confounds. The inclusion of 20 cats with chronic kidney disease (CKD) is a known potential clinical confound: on its own cats with CKD do not have significantly different serum artemin concentrations (Supplementary Figure S3) as compared with healthy cats. Running a multivariate correlation study showed that the presence of CKD did have a small effect on serum artemin concentrations (Supplementary Figure S4). Future studies with larger, more isolated cohorts will be needed to fully examine the influence of renal function on Artemin/GFRA3 Signaling. Furthermore, obtaining DRG from cats is a difficult process that is hampered by the availability of cadavers, a process which is further hampered by the difficulty of scheduling and obtaining radiographs prior to DRG isolation in cats. In addition, our DJD cat group was comprised of significantly older cats it is therefore possible that any differences seen in gene expression levels are simply due to natural aging in cats, and not due to the presence of DJD. Therefore, linear regression analyses were performed to determine the relationship between age and qRT-PCR expression and only found a significant correlation between age and expression of MRGPRD (Supplementary Table S1). Finally, it should be noted that all correlations could be spurious due to the limited sample size; therefore, larger longitudinal studies will be required to confirm these results.

## Conclusion

Our study provides robust mechanistic and systemic evidence linking the Artemin/GFRA3/TRP axis to chronic pain pathology in feline Degenerative Joint Disease (DJD). These data confirm that TRP channel expression are conserved in cats and artemin could act as a potential systemic biomarker for radiographic DJD severity. These findings validate the naturally occurring feline DJD as a highly relevant translational model for osteoarthritis and identify these upregulated TRP channels as promising, novel targets for developing effective therapeutic pain interventions in both veterinary and human medicine. Finally, these results demonstrate that cats could serve as a useful pre-clinical model for human DJD given the high prevalence of DJD in cats.

## Data Availability

The raw data supporting the conclusions of this article will be made available by the authors, without undue reservation.

## Supplementary material

The Supplementary Materialfor this article can be found online at: https://www.frontiersin.org/articles/10.3389/fpain.2026.1716651/full#supplementary-material
