# Supplementary material for: Artemin/GFRA3 axis and TRP channels: molecular insights from a feline model of osteoarthritis
Source: Front Pain Res (Lausanne). 2026 Mar 17;7:1716651. doi: 10.3389/fpain.2026.1716651 (PMC13036214; doi:10.3389/fpain.2026.1716651)
Supplement: Supplementary file 1 [file Datasheet1.pdf]

Supplementary Figures and Table

Artemin/GFRA3 Axis and TRP channels: Molecular Insights from a Feline Model of Osteoarthritis

Joshua J. Wheeler[1,2], Chie Tamamoto-Mochizuki[1,3,4], Margaret Gruen[5], Duncan Lascelles[5,6,7,8,9*], Santosh K. Mishra[1,2,7*]

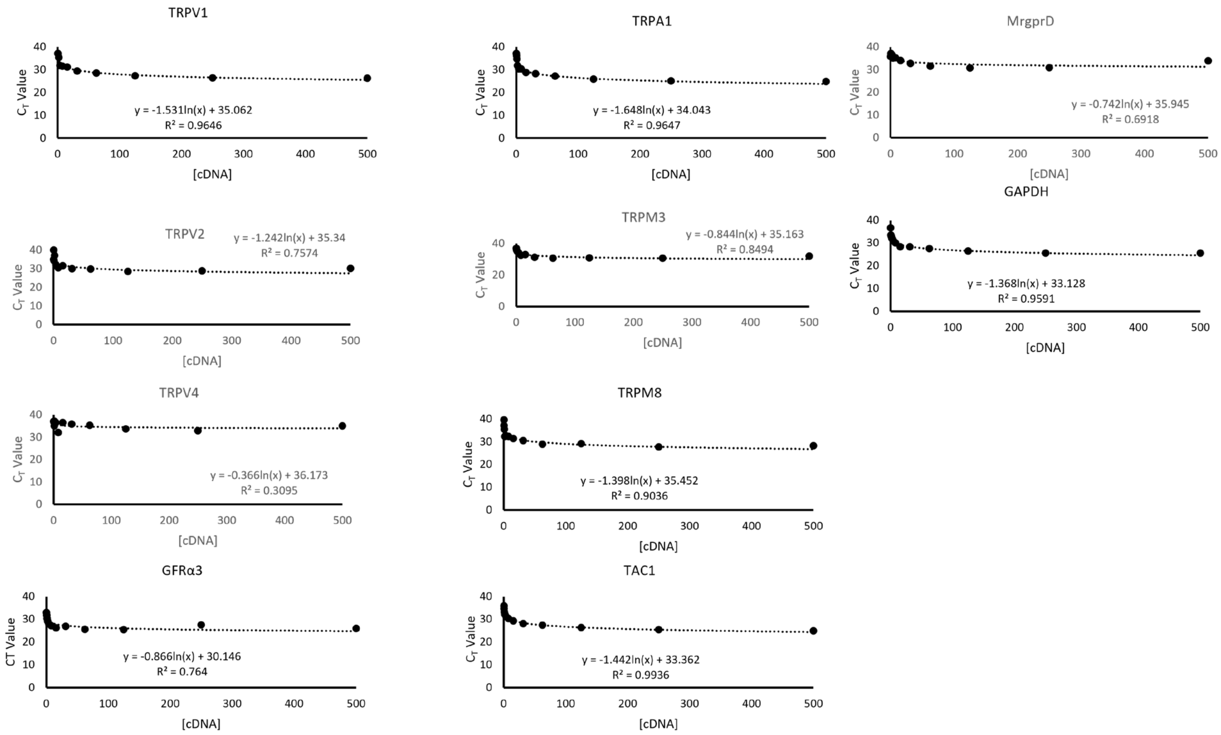

Supplementary Fig. S1: Pfaffl Calculation curves used to generate primer pair efficiencies. Standard curves used to generate primer efficiency values, E, used to determine the ratio of each gene in DRG from DJD versus Non-DJD cats.

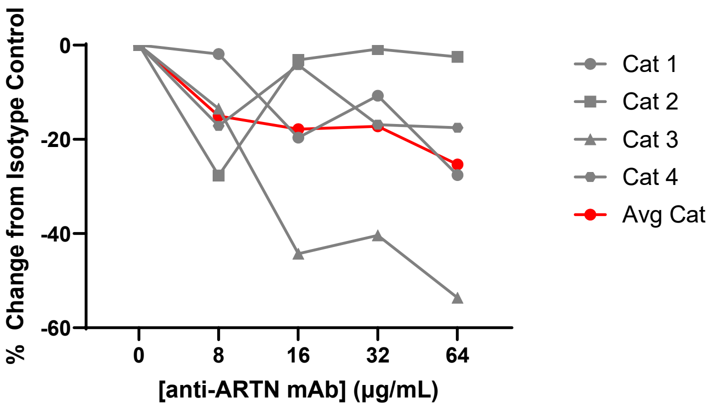

Supplementary Fig. S2: Validation that the hARTN ELISA kit can detect cat ARTN. Increasing concentration of anti-ARTN antibodies causes a concomitant decrease in the amount of cat ARTN detected by the human-specific ARTN ELISA kit.

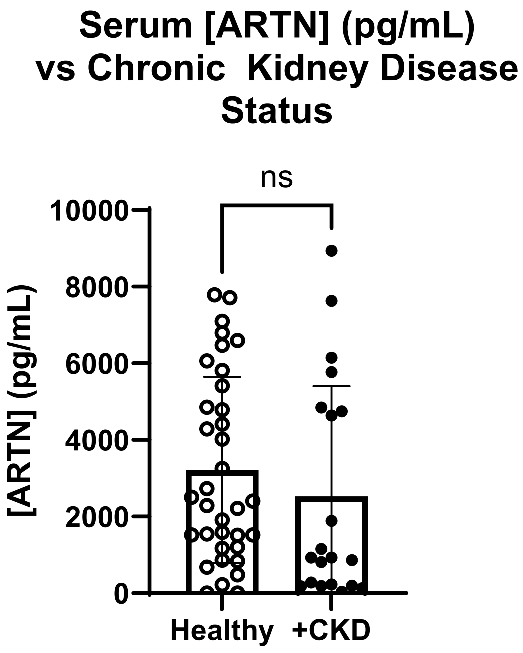

Supplementary Fig. S3: No significant differences were seen between serum artemin concentrations in healthy cats and cats with chronic kidney disease (CKD). Data is presented as Mean ± Standard Deviation. Each dot represents one biological replicate. Significance was determined using a Mann-Whitney U-test, ns = not significant.

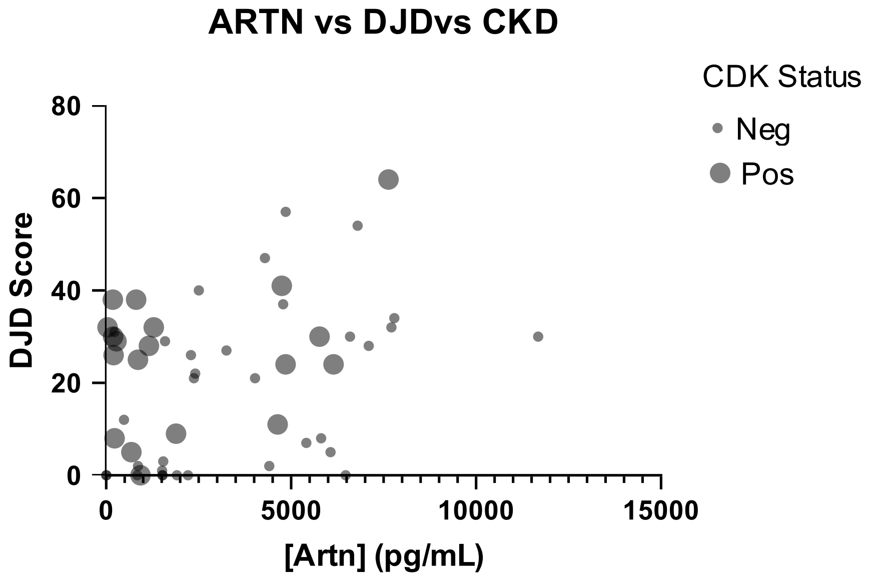

Supplementary Fig. S4: The presence of CKD likely results in lower serum artemin concentrations when added as a second variable with DJD score. When added as a second variable with DJD score, it becomes a weak, but significant predictor (*p = 0.0110, R^2 = 0.0484), of serum artemin concentrations. Note that CKD positive cats tended to have *lower* serum artemin concentrations. Significance was determined using a Multiple Linear Regression model; R^2 = 0.223 for the whole model.

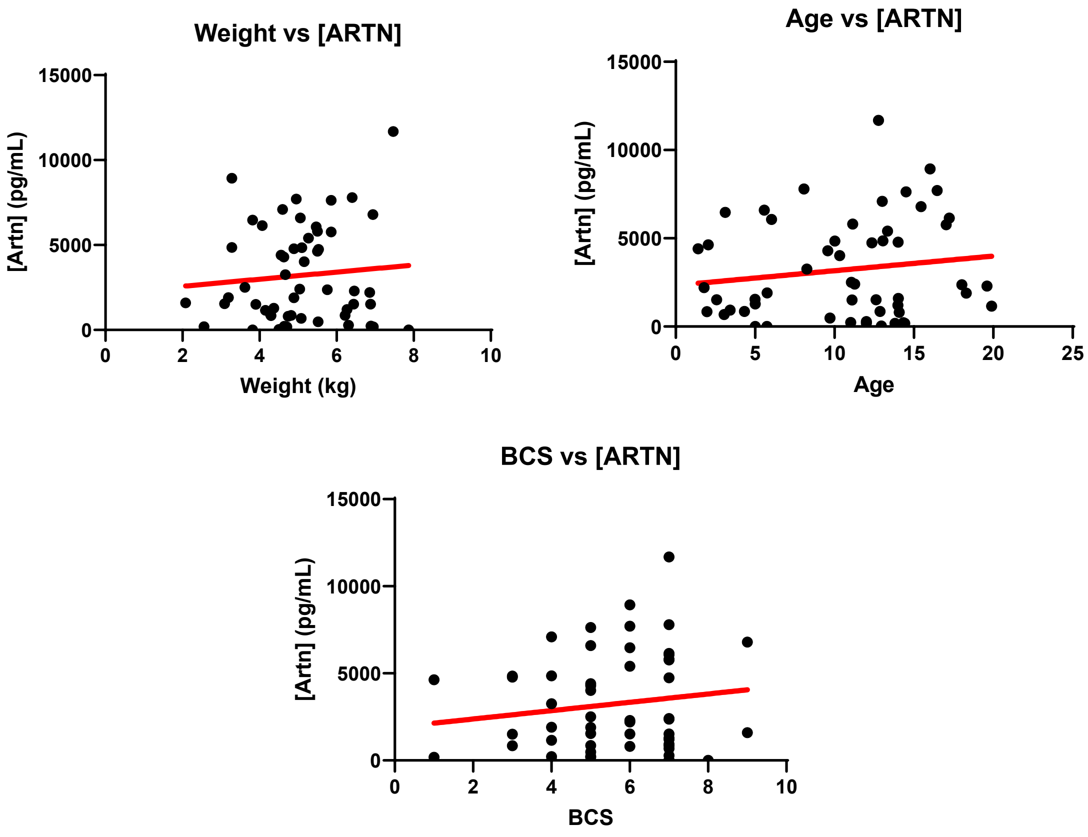

Supplementary Fig. S5: Serum artemin concentrations were not significantly correlated with cat weight (in kg), age, or body condition score (BCS). Each Dot represents one biological replicate. Significance was determined using a simple linear regression in Graphpad Prism.

	BCS[1]	Weight (kg)	X-ray Score	Age	Health[2]	Gender[3]
TRPV1	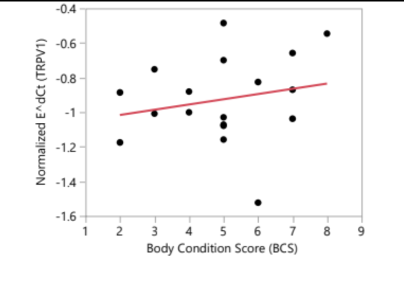	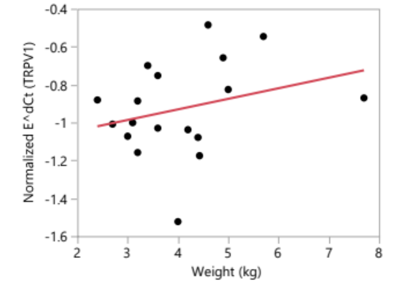	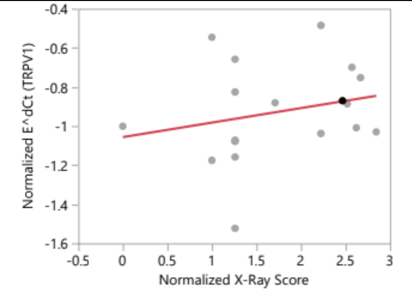	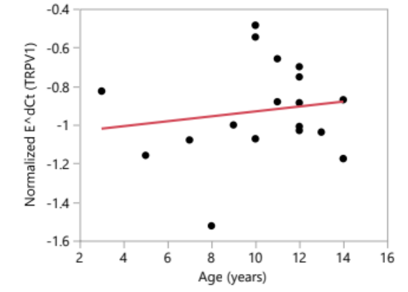	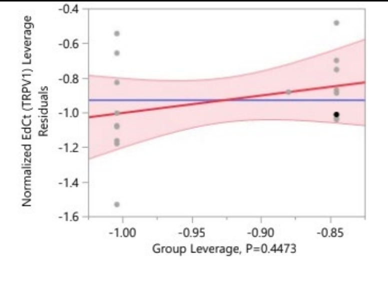	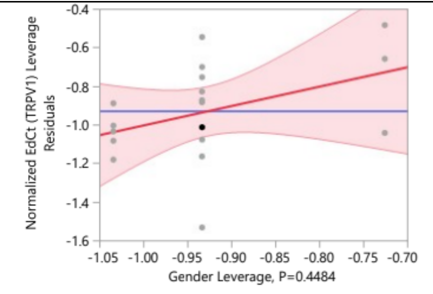
	$R^2 = 0.0439$; $R^2_{Adj} = -0.0159$	$R^2 = 0.0791$; $R^2_{Adj} = 0.0216$	$R^2 = 0.0551$; $R^2_{Adj} = -0.0039$	$R^2 = 0.0228$; $R^2_{Adj} = -0.0383$	$R^2 = 0.10$; $R^2_{Adj} = -0.02$	$R^2 = 0.17$; $R^2_{Adj} = -0.01$
	$p = 0.4042$	$p = 0.2581$	$p = 0.3484$	$p = 0.5497$	$p_{DJD} = 0.5564$<br>$p_{Healthy} = 0.3871$	$p_F = 0.8083$<br>$p_{FS} = 0.1538$<br>$p_M = 0.7837$<br>$p_{Total} = 0.4484$
TRPV2	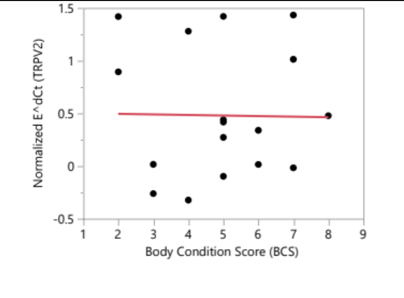	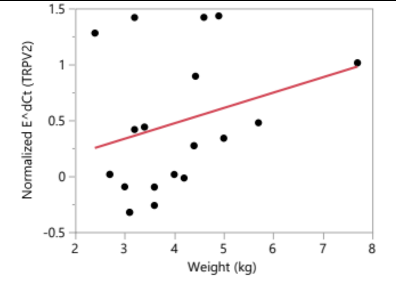	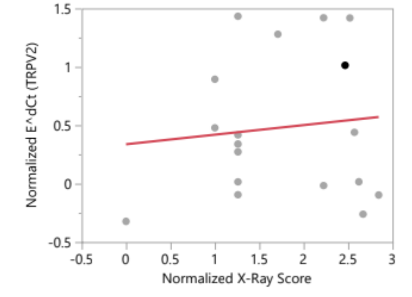	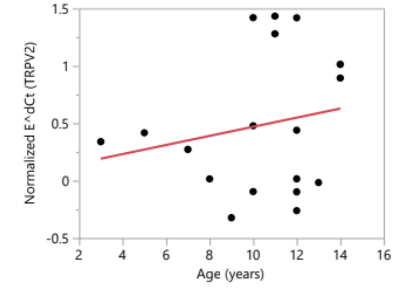	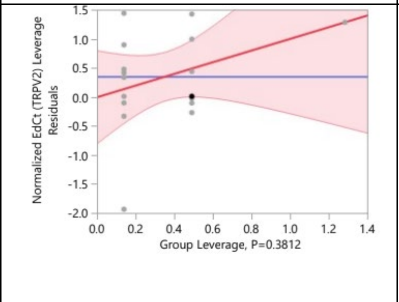	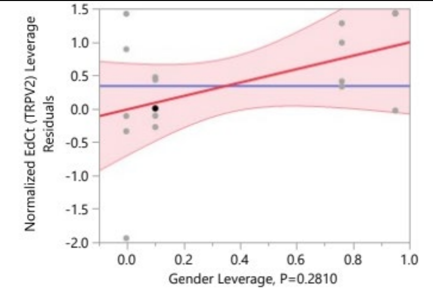
	$R^2 = 0.000232$; $R^2_{Adj} = -0.0623$	$R^2 = 0.0802$; $R^2_{Adj} = 0.0227$	$R^2 = 0.0112$; $R^2_{Adj} = -0.0505$	$R^2 = 0.0370$; $R^2_{Adj} = -0.0232$	$R^2 = 0.12$; $R^2_{Adj} = 0.003$	$R^2 = 0.23$; $R^2_{Adj} = 0.07$
	$p = 0.9522$	$p = 0.2549$	$p = 0.6754$	$p = 0.4448$	$p_{DJD} = 0.6820$<br>$p_{Healthy} = 0.1723$	$p_F = 0.3877$<br>$p_{FS} = 0.2145$<br>$p_M = 0.2671$<br>$p_{Total} = 2810$
TRPV4	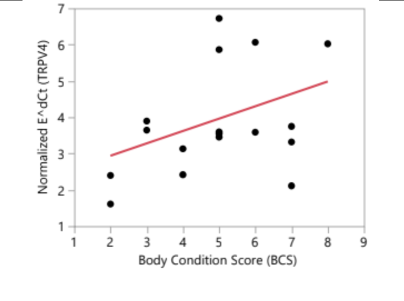	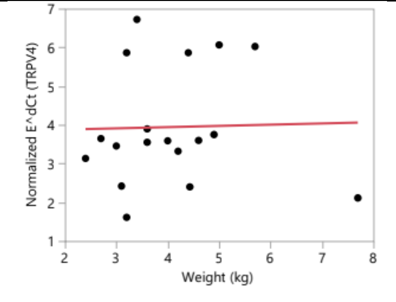	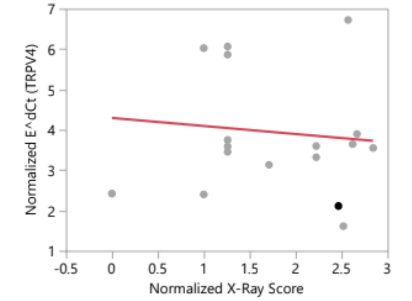	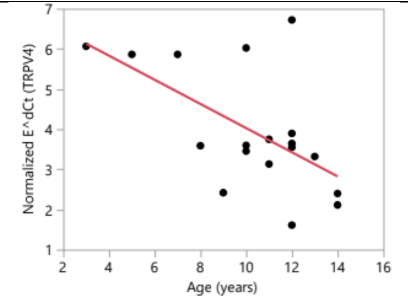	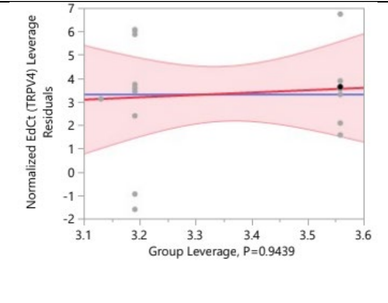	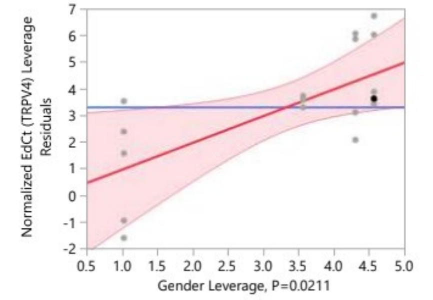
	$R^2 = 0.151$; $R^2_{Adj} = 0.0979$	$R^2 = 0.00069$; $R^2_{Adj} = -0.0618$	$R^2 = 0.0108$; $R^2_{Adj} = -0.0510$	$R^2 = 0.344$; $R^2_{Adj} = 0.303$	$R^2 = 0.0077$; $R^2_{Adj} = -0.12$	$R^2 = 0.49$; $R^2_{Adj} = 0.38$
	$p = 0.1111$	$p = 0.9173$	$p = 0.6815$	**$p = 0.0105$**	$p_{DJD} = 0.7906$<br>$p_{Healthy} = 0.9173$	$p_F = 0.2242$<br>$p_{FS} = 0.8118$<br>$p_M = 0.852$<br>**$p_{Total} = 0.0211$**

TRPA1	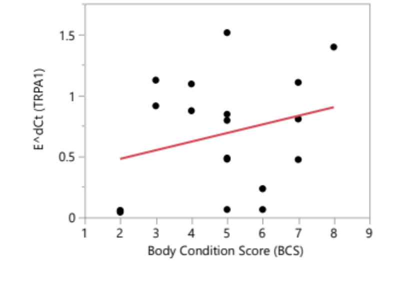	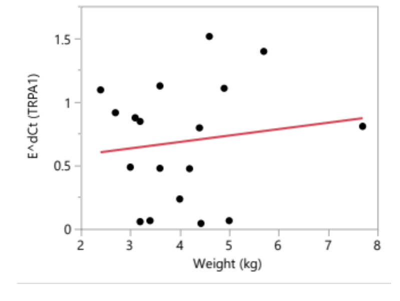	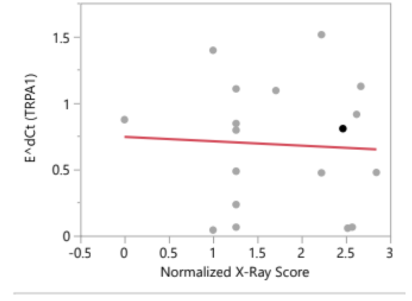	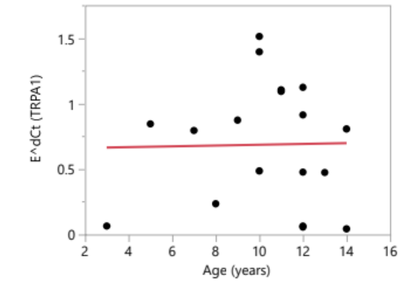	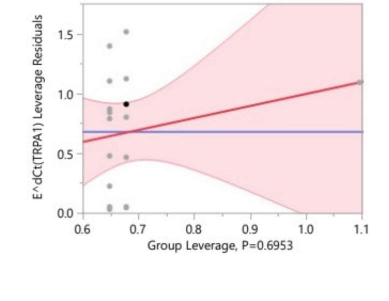	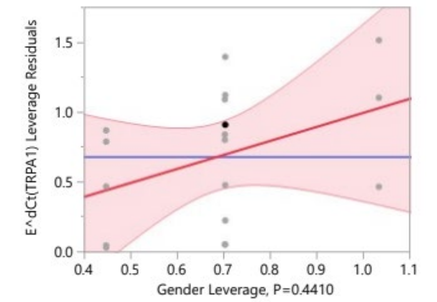
	R^2 = 0.0674 R^2_{Adj} = -0.00914	R^2 = 0.0183 R^2_{Adj} = - 0.0430	R^2 = 0.00308 R^2_{Adj} = - 0.0592	R^2 = 0.000369 R^2_{Adj} = - 0.0621	R^2 = 0.047 R^2_{Adj} = - 0.08	R^2 = 0.17 R^2_{Adj} = - 0.008
	p = 0.2981	p = 0.5921	p = 0.8267	p = 0.9397	p_{DJD} = 0.5450 $p_{Healthy}$ = 0.4518	p_F = 0.9265 p_{FS} = 0.1901 p_M = 0.9200 p_{Total} = 0.4410
TRPM3	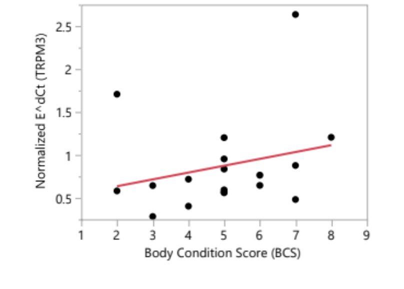	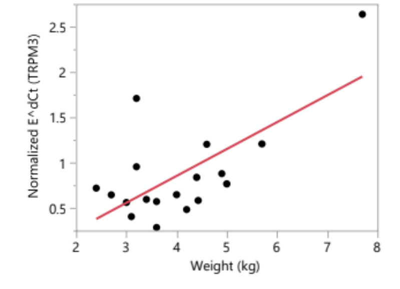	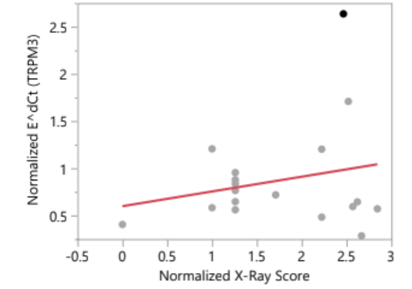	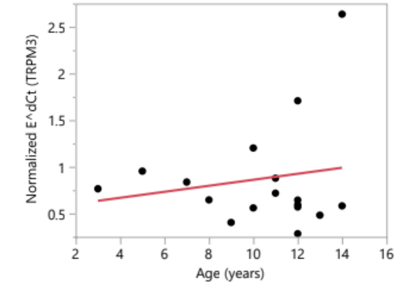	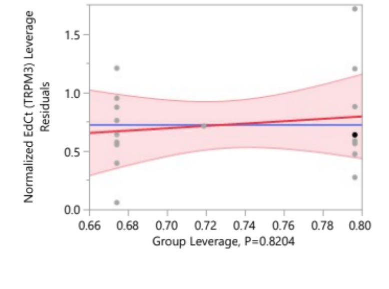	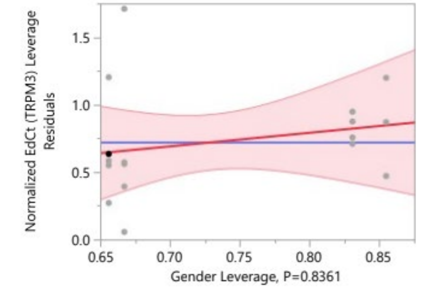
	R^2 = 0.0612 R^2_{Adj} = 0.00252	R^2 = 0.459 R^2_{Adj} = -0.421	R^2 = 0.0495 R^2_{Adj} = - 0.010	R^2 = 0.0297 R^2_{Adj} = - 0.0310	R^2 = 0.026 R^2_{Adj} = - 0.10	R^2 = 0.057 R^2_{Adj} = - 0.14
	p = 0.3223	**p = 0.0021**	p = 0.3750	p = 0.4943	p_{DJD} = 0.6978 $p_{Healthy}$ = 0.7422	p_F = 0.6587 p_{FS} = 0.6024 p_M = 0.5399 p_{Total} = 0.8361
TRPM8	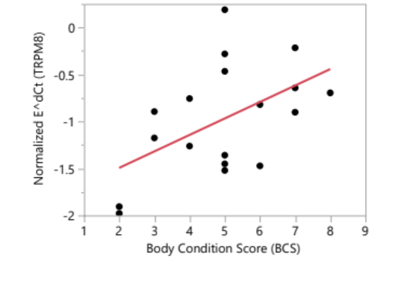	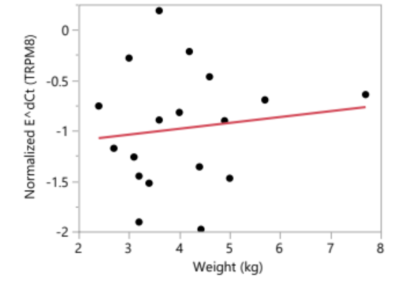	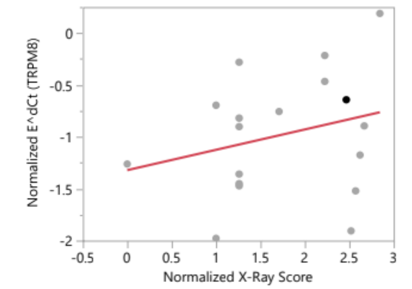	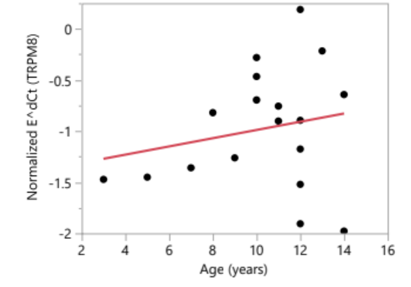	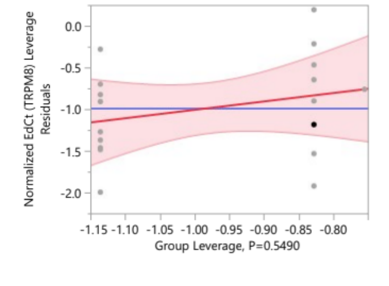	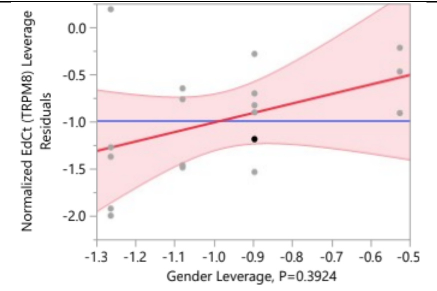
	R^2 = 0.267 R^2_{Adj} = 0.221	R^2 = 0.0156 R^2_{Adj} = -0.0459	R^2 = 0.0703 R^2_{Adj} = 0.0122	R^2 = 0.0414 R^2_{Adj} = -0.0185	R^2 = 0.077 R^2_{Adj} = - 0.05	R^2 = 0.19 R^2_{Adj} = 0.012
	**p = 0.028**	p = 0.6210	p = 2875	p = 0.4180	p_{DJD} = 0.7632 $p_{Healthy}$ = 0.3764	p_F = 5898 p_{FS} = 0.1574 p_M = 0.8420 p_{Total} = 0.3924

MRGPRD	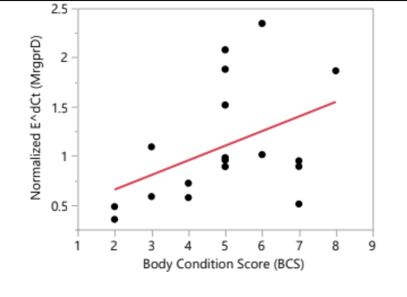	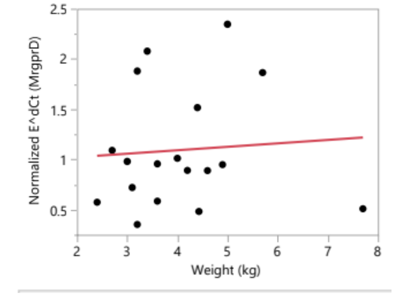	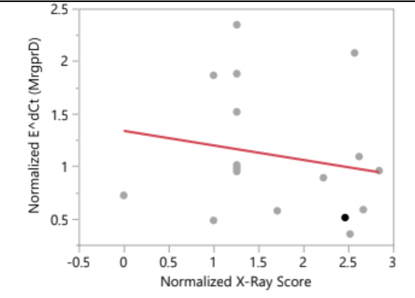	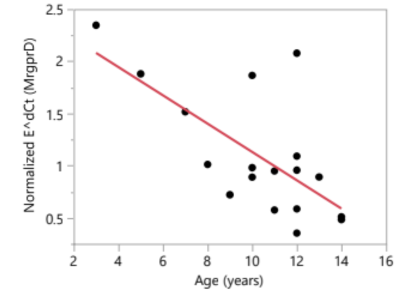	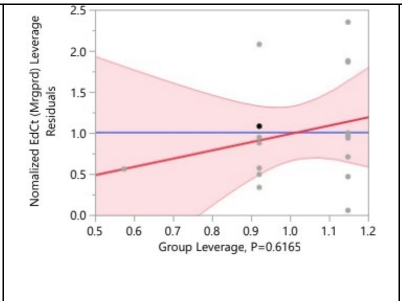	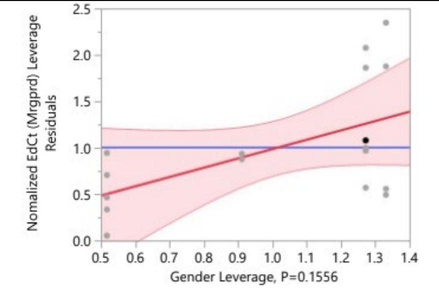
	$R^2 = 0.188$ | $R^2_{Adj} = 0.138$	$R^2 = 0.00534$ | $R^2_{Adj} = -0.0568$	$R^2 = 0.0342$ | $R^2_{Adj} = -0.026$	$R^2 = 0.458$ | $R^2_{Adj} = 0.424$	$R^2 = 0.062$ | $R^2_{Adj} = -0.06$	$R^2 = 0.30$ | $R^2_{Adj} = -0.15$
	p = 0.0719	p = 0.7733	p = 0.4626	**p = 0.0020**	p_{DJD} = 0.8872<br>$p_{Healthy}$ = 0.3421	p_F = 0.2186<br>p_{FS} = 7313<br>p_M = 0.2497<br>p_{Total} = 0.1556
TAC1	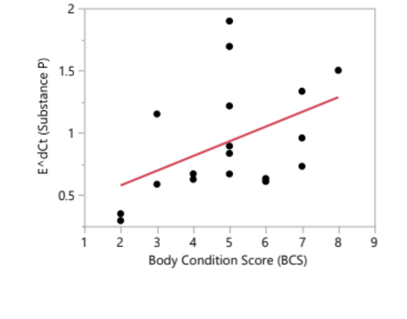	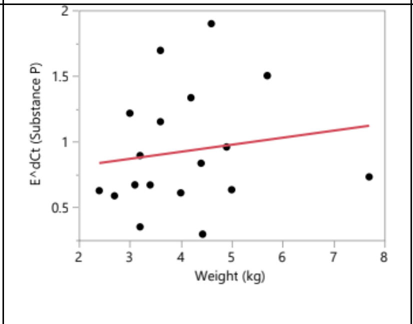	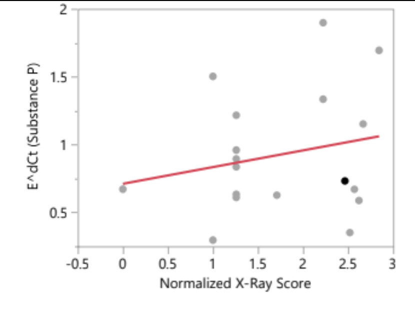	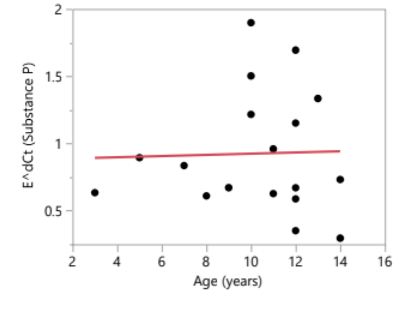	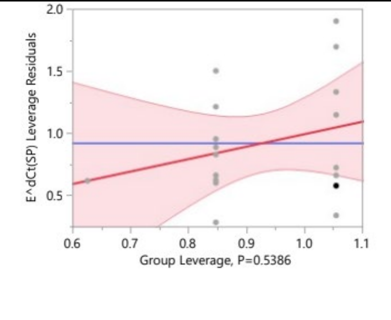	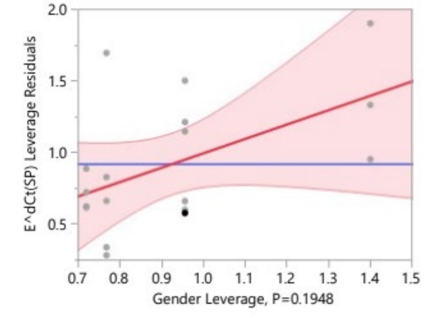
	$R^2 = 0.205$ | $R^2_{Adj} = 0.155$	$R^2 = 0.0226$ | $R^2_{Adj} = -0.0385$	$R^2 = 0.0467$ | $R^2_{Adj} = -0.013$	$R^2 = 0.00088$ | $R^2_{Adj} = -0.0616$	$R^2 = 0.079$ | $R^2_{Adj} = -0.04$	$R^2 = 0.28$ | $R^2_{Adj} = 0.12$
	p = 0.0594	p = 0.5513	p = 0.3891	p = 0.9070	p_{DJD} = 0.2976<br>$p_{Healthy}$ = 0.9810	p_F = 0.2081<br>**p_{FS} = 0.0478**<br>p_M = 0.9758<br>p_{Total} = 0.1948
GFRA3	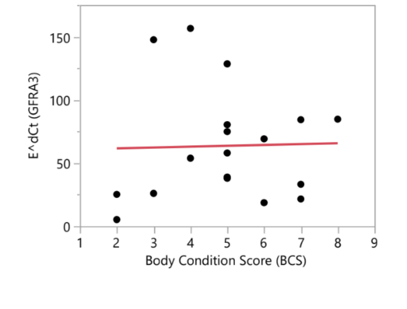	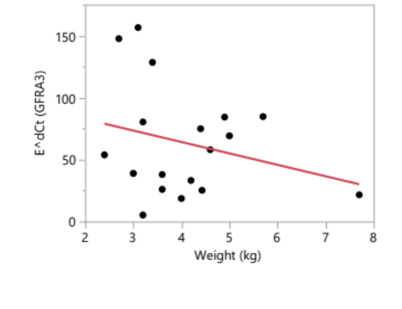	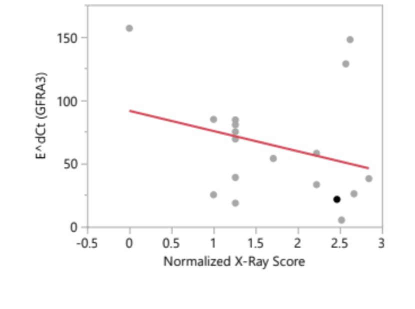	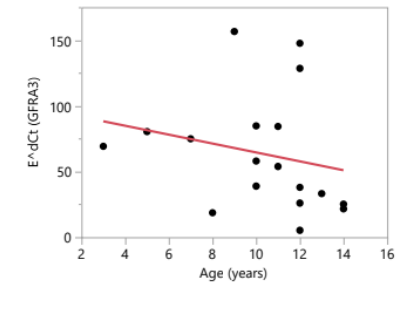	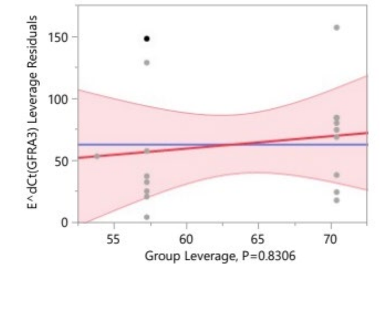	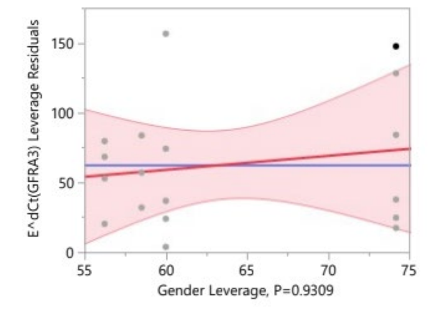
	$R^2 = 0.00071$ | $R^2_{Adj} = -0.0617$	$R^2 = 0.0683$ | $R^2_{Adj} = 0.0101$	$R^2 = 0.0807$ | $R^2_{Adj} = 0.0232$	$R^2 = 0.0511$ | $R^2_{Adj} = -0.0082$	$R^2 = 0.024$ | $R^2_{Adj} = -0.1$	$R^2 = 0.030$ | $R^2_{Adj} = -0.18$
	p = 0.9164	p = 0.2947	p = 0.2533	p = 0.3670	p_{DJD} = 0.8735<br>$p_{Healthy}$ = 0.6216	p_F = 0.7780<br>p_{FS} = 0.8744<br>p_M = 0.5258<br>p_{Total} = 0.9309

Supplementary Table 1 - Correlations between mRNA expression of nocifensive-associated genes and factors that can affect DJD status. R^2, R^2_{Adj}, and p-values determined using JMP. Graphs were generated in JMP p – values in highlighted in red are considered significant at α = 0.05. Raw E^dCt were calculated using equation 1 and normalized when necessary. [1]Body Condition Score, [2]Healthy or with DJD, [3] Male (M), Female (F), Male Castrated (MC), or Female Spayed (FS).
